# Estimating developmental states of tumors and normal tissues using a linear time-ordered model

**DOI:** 10.1186/1471-2105-12-53

**Published:** 2011-02-11

**Authors:** Bo Zhang, Beibei Chen, Tao Wu, Zhenyu Xuan, Xiaopeng Zhu, Runsheng Chen

**Affiliations:** 1 Laboratory of Bioinformatics and Noncoding RNA, Institute of Biophysics, Chinese Academy of Sciences, Beijing 100101, PR China; 2 Graduate University of Chinese Academy of Sciences, Beijing, 100037, PR China; 3 Department of Molecular and Cell Biology, Center for Systems Biology, University of Texas at Dallas. 800 W Campbell Road, Richardson, TX 75080, USA

## Abstract

**Background:**

Tumor cells are considered to have an aberrant cell state, and some evidence indicates different development states appearing in the tumorigenesis. Embryonic development and stem cell differentiation are ordered processes in which the sequence of events over time is highly conserved. The "cancer attractor" concept integrates normal developmental processes and tumorigenesis into a high-dimensional "cell state space", and provides a reasonable explanation of the relationship between these two biological processes from theoretical viewpoint. However, it is hard to describe such relationship by using existed experimental data; moreover, the measurement of different development states is also difficult.

**Results:**

Here, by applying a novel time-ordered linear model based on a co-bisector which represents the joint direction of a series of vectors, we described the trajectories of development process by a line and showed different developmental states of tumor cells from developmental timescale perspective in a cell state space. This model was used to transform time-course developmental expression profiles of human ESCs, normal mouse liver, ovary and lung tissue into "cell developmental state lines". Then these cell state lines were applied to observe the developmental states of different tumors and their corresponding normal samples. Mouse liver and ovarian tumors showed different similarity to early development stage. Similarly, human glioma cells and ovarian tumors became developmentally "younger".

**Conclusions:**

The time-ordered linear model captured linear projected development trajectories in a cell state space. Meanwhile it also reflected the change tendency of gene expression over time from the developmental timescale perspective, and our finding indicated different development states during tumorigenesis processes in different tissues.

## Background

Cancer is a severe threat to human health. Although there are many established methods for overcoming this disease, the high mortality caused by cancer is still a severe threat to human. Meanwhile, the side-effects of many therapeutic methods greatly affect the quality of life of individuals and their families. Uncertainty about the mechanisms of tumorigenesis greatly handicaps the creation and application of suitable therapeutic methods. Tumorigenesis is a complex process, affected by both genetic factors and environmental conditions. There is evidence to suggest that developmental processes and tumorigenesis share some conserved mechanisms [1,2]. Time-course microarray experiments have the advantage of allowing us to study the dynamics of gene regulation. Time-course microarrays have recently been used to identify biological markers associated with disease and to examine the expression patterns of genes that are important in tumorigenesis and development [1,3].

Many models have been proposed to explain the process of tumorigenesis and its relationship to development. The "cancer attractor" model was first suggested by Kauffman in the 1970 s [4] and can be used to explain how a Gene Regulation Network (GRN) confers a single genome with the capacity to produce a diversity of stable, discretely distinct cell types over the process of development [5]. Foster introduced a simplified differential equation described by Huang [6] into a model containing two genes. Five hundred "cells" were stimulated to "differentiate", finally reaching the "stable attractors" position, demonstrating the validity of the "cancer attractor" model. There is a significant amount of evidence based on time-course microarray experiments which supports the attractor theory [5,7-9]. Mar and Quackenbush [10] have recently decomposed cell fate transition into two processes: the core process that includes the main differentiation pathway, and a transient process that captures information from the environment and controls the core process.

Cell state space is a high-dimensional space in which different cell types correspond to points or distributions [11]. In Foster's work [5] a system based on two genes generated 3-dimensional coordinates including two gene dimensions and one "quasi potential" dimension, however, that still exists some difficulties to explain the biological meaning of this "quasi potential" dimension.. Since time is invariable and irreversible, sequentially ordered developmental progression is a very important innate characteristic of life. If we treat time as a scale for measuring cell state space, it is possible to describe the high-dimensional cell state space by a low-dimensional space.

Many approaches, including PCA and SVD methods [12-14], the Bayesian models [15], HMM(Hidden Markov Models) [16], and some ANOVA and regression-based model [17,18] have been applied for the analysis of time-course microarray data from different aspects. Most of these methods are designed to detect genes which undergo significant changes and to classify expression patterns in time-course experiments. Only few methods emphasize temporal order within experiments and time-course expression profiles.

Here, in order to capture the temporal properties and describe the trajectories of development processes, we propose a new linear model, named the "time-ordered linear model", which draws on the idea that a co-bisector can represent the main tendency of a series of vectors. This co-bisector model has two main advantages: first, unlike present methods such as PCA, the biological meaning of the co-bisector model is borne in mind in the design of the model. A co-bisector conserves the temporal properties of a series of vectors since they have order-restricted projection locations on the co-bisector. Furthermore, our model preserves the spatial distance ratio between neighboring samples which have fixed locations in microarray space. Our time-ordered linear model can be used as a measurement scale of gene expression variation in microarray space, thus creating a new application for time-course microarray data; estimating the expression pattern similarities between expression data from more than one source. In the present work, we apply our time-ordered linear model to estimate expression pattern similarities between different tumor tissues and their corresponding normal tissues in both mice and humans. Our time-ordered linear model describe the trajectories of development process in a cell state space from the gene expression pattern perspective, thus helping us to improve our understanding of the relationships among different cell types in cell state space.

## Results

### Design the Time-ordered Linear Model in the Abstract Cell State Space

The concept of cell state space was proposed by Kauffman[11]. In high-dimensional cell state space, cell types with similar properties are grouped together. The dimensions of cell state space are measurements of cell properties such as SNP, transcriptome, and epigenetic modification. The expression pattern of a cell is simply a reflection of its cell state. Recently, the mouse and human genome DNA methylation maps have been reported [19,20]. We believe that a fuller and more detailed description of cell state space will emerge as more and more high-throughput data are published. But the work presented here only focus on gene expression patterns, and we simplified the cell state space as a microarray space which dimensions are determined by genes.

**Figure 1:**
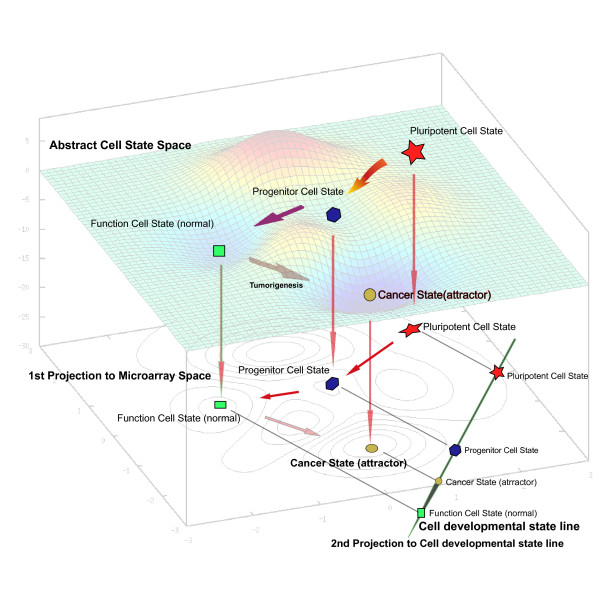
**Development trajectory in Cell State Space and transformations to cell developmental state line in a sub space**. In cell status space, the development process is represented as a segment of line; the experimental samples are selected to represent time points in this continuous segment. After experimental detection such as microarray hybridization or proteins 2D-eletrophoresis, the experimental samples have transformed to points in the sub-attribute space (microarray space or proteomics space). The time-ordered linear model projects the samples points to the cell developmental state line which keeps the strict temporal order of every sample and conserves distance ratio between neighbor points.

Since all cell activities are continuous, any cellular process can be represented as a continuous thread in abstract cell state space. In Figure 1 the process of cell differentiation, from the pluripotent to the differentiated cell state, is described as a continuous track in abstract cell state space. When microarrays are used to describe the transcriptome, the expression pattern is projected from abstract cell state space to microarray space, and the continuum of cellular processes is retained and can be used to map cell differentiation in microarray space.

In cell differentiation microarray experiments, samples representing different cell differentiation stages i.e. different time points, are linked in order by a curve in microarray space. As a powerful approach, the PCA method can easily draw the mathematical distribution of principal components of these points to maximize the sum of variance. In order to test the efficiency of description development trajectory by PCA methods, we analyzed dataset GSE13149 which represents mouse fetal liver development. Although some principal components (PCs) may preserve the sampling order (Figure S1), one hand, the biological meaning of these PCs can not be derived directly from the PCA methods. Some approaches, such as GO annotation, were applied to obtain the biological meaning of these PCs from the biological function of significantly changed genes. On the other hand, under the condition that maximizes sum of variance, the projection distance ratio of neighbor points on PCs are different from real distance ratio in microarray space (Table S1). For these two reasons, it is difficult to use the PCA method to describe the trajectories of the development process.

**Figure 2:**
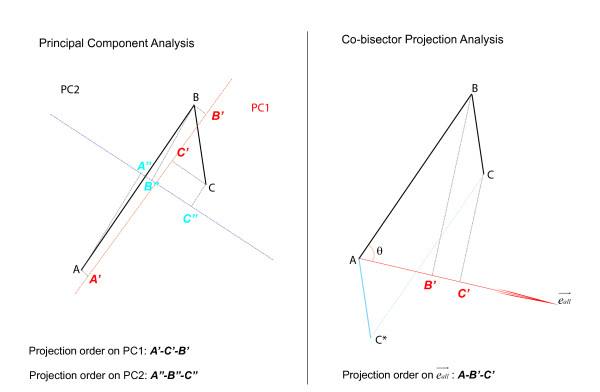
**Difference between PCA and Time-ordered linear model**. Vector $\vec{AB}$ and $\vec{BC}$ existing in a 2-D space, represent a cell departed form state A, bypassing state B, finally reached to state C. Left: Principal component analysis generates two features, PC1 corresponds to a line that passes through the mean and minimizes sum squared error; PC2 is perpendicular to PC1. On PC1 and PC2, the order of two vectors may be lost. Right: Time-ordered linear model generates one cell state line $\vec{e_{all}}$. On this line, the order of two vectors are preserved perfectly, meanwhile, the distance ratio is also preserved: $\vec{AB'}:\vec{B'C'}=\vec{AB}:\vec{BC}$.

Since this curve is difficult to describe in high-dimensional space, we have to develop a time-order linear method to characterize this curve using a simple line, preserving the order of sampling points in the cell differentiation process. Different to PCA method (Figure 2), our linear model is designed to describe the development trajectory as a line with a distinct direction, which represents the change in genes expression over developmental time. In microarray space, when a series of points (expression profiles of samples in experiments) are projected onto a line, the sampling order of the projected points are preserved; meanwhile, if the projection distance ratio of neighbor sample points on the line are equal to the distance ratio of neighbor samples points in the microarray space, we can say this line reflect the change of genes expression over time. Naturally, we found that the angle bisector linear model would satisfy these two conditions. Since feature reduction involves loss of information, we maximized the distance between points on the bisector in order to preserve as much information as possible. The details of model construction were described in method.

Using this time-ordered linear model we can obtain "cell developmental state lines" representing the temporal properties of differentiation trajectories. The main advantages of this time-ordered model are that the sequential order is preserved and that the distance between points is maximized, ensuring that developmental processes are in the right order and that the relationship among neighboring points in microarray space is denoted accurately. Here, by analyzing published tissue development and cell differentiation expression profiles obtained using time-course experiments (described below), we obtained cell developmental state lines representing several developmental and differentiation processes, and then compared published tumor expression profiles obtained using the same microarray platform (described below). By calculating the projection positions of expression profiles on the cell developmental state line, and their relationship, we were able to deduce developmental states of these tumors in these processes on a developmental-temporal scale.

Similar to PCA, Time-ordered linear model generate one principal component to represent a mass of sample points. This principal component which we called 'developmental state line', is not only representing one mathematical characteristic of samples, but also reflects temporal property of a development process. In other words, the biological meaning was denoted to a mathematical characteristic, and this clear biological development perspective enable us to accurately estimate different development stage of same tissues samples came from different sources by one line.

**Figure 3:**
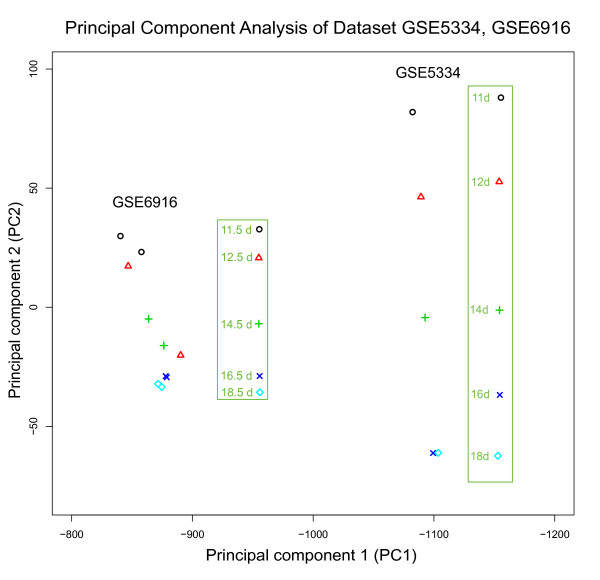
**Principal Component Analysis of Dataset GSE5334 and GSE6916**.

**Figure 4:**
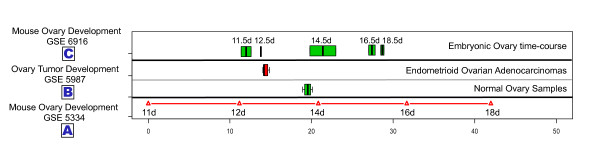
**Mouse fetal ovary cell developmental state line distinguished temporal distributions of mouse ovary tissue and cancer**. (A) Projection positions of Mouse fetal ovary development time-course sample on Cell developmental state line based on dataset GSE 5334. The sample 11 d was set as origin point; other projection positions were normalized by sample 11 d. (B) Distributions of projection positions of ovary samples from Trim-28 Knockout mouse and normal WT mouse (dataset GSE5987). Green represents normal WT mouse ovary samples. Red represents Endometrioid ovarian adenocarcinomas samples from Trim-28 Knockout mouse. (C) Distributions of projection positions of ovary development time-course experiments based on GSE6916.

In order to verify whether the principal components generated by PCA method have the same property, we used PCA to analyze dataset GSE5334 which contains mouse ovary development time-course expression profiles, then projected another development time-course data GSE6916 to principal components (PCs) of GSE5334. The result was shown in Figure 3. PC1 reserved the natural time order of samples in GSE5334, but can not estimate the right order of samples in GSE6916. PC2 can not keep the right natural time order of 16 day and 18 day in ovary development expression profiles of GSE5334. In GSE6916, PC2 mainly kept the right order of development except that sample of 12.5 day was project between 14.5 day and 16.5 day. Oppositely, the cell development state line of GSE5334 kept the right natural development order of samples both in GSE5334 and GSE6916 (Figure 4). We also calculated the distance and distance ratio between neighbors points (Table 1). Only cell development state line generated by Time-order linear model can keep the right distance ratio information which existed in high dimensional microarray space. We believe the Time-ordered linear model is suitable to describe the development trajectory in a microarray space and estimate the developmental stage of samples came from other experiments.

**Table 1:** Distance and Distance Ratio in microarray Space, developmental state line (DSL) and PCs of dataset GSE5334

	11d->12d	12d->14d	14d->16d	16d->18d
Distance in microarray space (i->i+1)	118.62	105.49	114.97	104.52
Distance ratio in microarray space ((i->i+1): (i+1->i+2))		**1.12**	**0.92**	**1.10**

Distance on DSL (i->i+1)	11.25	10.01	10.85	9.91
Distance ratio on DSL ((i->i+1): (i+1->i+2))		**1.12**	**0.92**	**1.10**

Distance on PC1 (i->i+1)	-5.91	-3.46	-6.55	-4.27
Distance ratio on PC1 ((i->i+1): (i+1->i+2))		**1.70**	**0.53**	**1.53**

Distance on PC2 (i->i+1)	-35.89	-50.62	-56.88	0.09
Distance ratio on PC2 ((i->i+1): (i+1->i+2))		**0.71**	**0.89**	**-627.75**

The robustness of this time-ordered linear model was also tested. We generated 3 cell developmental state lines by removing 1, 2 and 3 points from dataset GSE13149. Then dataset GSE6998 were projected to these 3 cell developmental state lines one by one. The results indicated that the projection locations of the test dataset GSE6998 maintained the order following interval of points of the cell development state lines. Meanwhile, the mean values of the projection locations were similar to the control, and the variance constantly increased. Especially the first test point 10.5 D and last test point 16.5 D, suffered bigger variance than other points (Table S2 and Figure S2). Interestingly, lack of the first two or three points influenced the resolution of later development period samples. This result indicated that the model has a high robustness, especially for the lack of medial samples, but lack of samples at either end of the time points would influence the projection result.

### Mouse liver cell developmental state line demonstrates apparent "similarity to early developmental stage" of liver tumors

**Figure 5:**
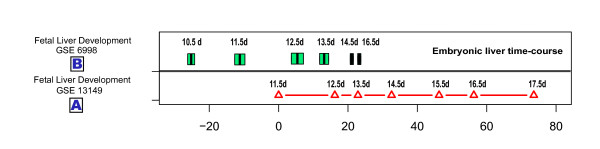
**Mouse fetal liver cell developmental state line**. (A) Projection positions of the mouse fetal liver development time-course sample on Cell developmental state line based on dataset GSE 13149. The sample 11.5 d was set as origin point; other projection positions were normalized by sample 11.5 d. (B) Distributions of projection positions of the CD-1 mouse liver samples from dataset GSE 6998.

The liver development "cell developmental state line" was calculated according to the method described above using dataset GSE13149, which traces mouse fetal liver development from 11.5 days to 18.5 days (Figure 5). Dataset GSE6998, another mouse liver development time-course expression dataset, was used to test the accuracy of this cell developmental state line. As shown in Figure 1B, the cell developmental state line could accurately order liver samples according to developmental stage. Compared to the cell developmental state line, the projection positions of dataset GSE6998 were earlier. This alteration might be caused by the use of different mouse strains in the datasets analyzed; the cell developmental state lines are based on C57/B6 mouse liver development, while the GSE6998 dataset came from experiments with CD-1 mice. Our results indicated that the cell developmental state line reliably reflects the temporal property of other expression datasets, and can be used to compare data generated from different experiments.

**Figure 6:**
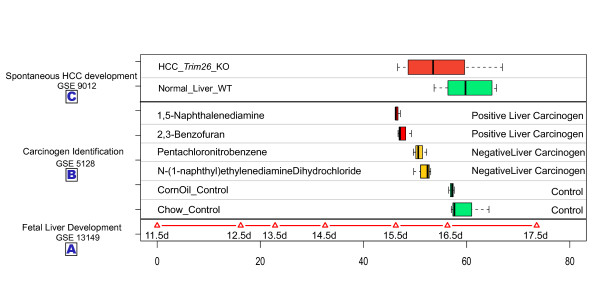
**Temporal distributions of mouse normal liver tissues and tumors**. (A) Projection positions of mouse fetal liver development time-course samples on the Cell developmental state line based on dataset GSE 13149. The sample 11.5 d was set as the origin point; other projection positions were normalized by sample 11.5 d. (B) Distributions of projection positions of mouse liver samples treated with carcinogens from dataset GSE 5128. Green represents normal mouse liver samples. Yellow represents liver samples from mice fed negative carcinogens. Red represents liver samples from mice fed with positive carcinogen for 13 weeks. (C) Distributions of projection positions of mouse HCC samples and normal WT liver samples based on dataset GSE9012. Green represents normal WT mouse liver samples. Red represents mouse HCC samples.

We used the liver cell developmental state line to estimate the similarities of liver tumor samples over time. Expression profiles of 10 liver tumor samples induced by knockout of Trim24 (Trim24-KO) and 5 normal samples from the dataset GSE9012 were individually projected onto the cell developmental state line as described above. Compared with normal samples, the expression patterns from tumor samples had a clear tendency to project to positions corresponding to earlier development stages (Figure 6). The projection positions of expression patterns of normal liver samples and tumor samples were 53.83 ± 9.42 and 59.55 ± 5.96, respectively. Such results demonstrate that the cell state of liver tumors induced by Trim24-KO was more similar to that of earlier stages of development, suggesting that knockout of Trim24 may block cell development.

We projected the dataset GSE 5128 which contains a series of carcinogen-treated samples onto the liver cell developmental state line. Interestingly, a dynamic back-moving tendency appeared (Figure 6). Mice treated with the two positive liver carcinogens had the earliest projection positions (those treated with 1,5-Naphthalenediamine located at46.52 ± 0.54, while those treated with 2,3-Benzofuran located at 47.70 ± 1.40). Projection positions for mice treated with the two negative liver carcinogens located in the middle between control and positive liver carcinogen-treated mice. These results suggest that there is a link between carcinogenicity and the apparent "younger developmental state" of cells observed here; different carcinogens change the cell state to different degrees. Our liver cell developmental state line represents the changes in liver cell state associated with development. The analysis of these two datasets suggested a fact that liver tumors have a similar cell state to that of earlier developmental stages of the fetal liver. As is well known, tumor or cancer cells have many of the properties of self-renewing stem cells. It is accepted that once the cell status of cancer cells departs from that of normal cells, they regain the ability to proliferate uncontrollably.

### Mouse ovary tumor demonstrates apparent "developmentally younger"

We conducted a similar analysis of ovary development time-course experimental data. In dataset GSE 5334, expression profiles from 5 stages (Gestational days GD11 day to GD18) were used to construct the ovary cell developmental state line as described above. Dataset GSE6916 was used to test the accuracy of the cell developmental state line. The results indicated that the cell developmental state line ordered each developmental stage accurately (Figure 4). We then used the cell developmental state line to estimate the projection positions of dataset GSE5987, which contains 7 ovarian tumors and 4 normal ovary samples. Results were also similar to those described above for the liver (Figure 4), once again suggesting that the process of tumorigenesis in ovary make ovary tumor cells have a high "similarity to early developmental stage".

### Cell state variation caused by carcinogenesis in the lung do no share same direction to mouse lung cell developmental state line

Three sets of expression data from different developmental stages were projected onto the lung cell developmental state line (based on dataset GSE11539 which represents lung development from embryonic day 11.5 to postnatal day 5) (Figure S3). The projection positions of each of the samples were distributed from early to terminal differentiated stages according to the age of the mice. This result demonstrates that the cell developmental state line can faithfully represent the temporal properties of development. Then the cell developmental state line was used to estimate the states of GSE5127 lung carcinogenesis expression dataset, in which different carcinogens were feed to the mouse. The results were different to those for the liver and ovary. The four chemicals hardly changed the "developmental state" of lung cell state (Figure S4). Although 2,3-Benzofuran and 1,5-Naphthalenediamine can cause cancer in both liver and lung, our model indicates that, different to in the liver and ovary, the genes belonged to a developmental pathway that may have not been involved in carcinogenesis process in the lung.

Generally speaking, the cell developmental state lines generated by our time-ordered linear model using time-course microarray experimental data accurately reflect the gene expression pattern variation over time during development, and their utility for estimating the relationship between tumor cell state and normal cell state could supply clues for further investigations of the tumorigenesis mechanism. If tissue-specific tumors are treated as "attractors", the cell developmental state line describes the relative position of the "attractors" in cell state space. In the liver and the ovary, cancer attractors may be located nearer to earlier developmental stages than is the case for normal tissues. In the lung, cancer attractors may be located in a direction that is vertical to the developmental direction, and the cell developmental state line can not distinguish such cell state changes. Projection results for cancer samples give an indication that the mechanisms of tumorigenesis may be not the same in different tissues.

### Mouse tissues development trajectories have different directions in cell state space

**Figure 7:**
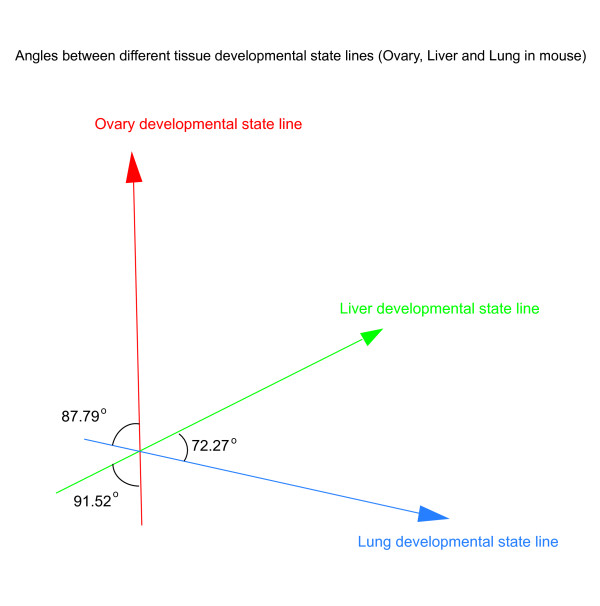
**Angles between different tissue development state lines**. **Red: **Mouse fetal ovary developmental state line (GSE5334); **Green: **Mouse fetal liver developmental state line(GSE13149); **Blue: **Mouse fetal lung developmental state line(GSE11539).

Initiating from a fertilized egg, more than 200 kinds of cell types are generated follow different differentiation trajectories in both mouse and human. After transforming mouse liver, ovary and lung tissue development trajectories to tissue developmental state lines, we calculated the angles of these three developmental state lines. Obviously, smaller angel between developmental state lines suggests higher similarity of gene expression pattern between two tissue development processes. The result shown in Figure 7 indicated the liver development and lung development shared more commons (angle of liver-lung is 72.27 degree), and the ovary development had a nearly vertical direction to liver and lung developmental state lines (angle of liver-ovary is 91.52 degree; angle of lung-ovary is 87.79 degree).

As known, the liver and lung both come from the endoderm, and the ovary is developed from mesoderm. We guess that if the origins of tissues in the gastrula are same, their development trajectories may share more commons in the cell state space, and the angle between these tissue developmental state lines would be smaller.

### Cell differentiation state lines of human ES cell distinguished cell states variation of human tumors

Since human embryonic stem cells are an important model for studying human development, we transformed expression data from ES cell differentiation time-course experiments to a cell developmental state line representing the ES cell differentiation process. Unlike mouse tissues developments start from different cell states, we calculated two cell developmental state lines for all ES cell differentiation processes starting from the same pluripotent cell state. This generated 2-dimensional coordinates, with each cell developmental state line axis representing different developmental processes. We used the GSE9940 dataset of the ES cell-derived neural rosette differentiation expression profile to generate a "neuronal" cell developmental state line, and the GSE8884 dataset of the ES cell-derived blast cell differentiation expression profile to generate a "blast cell" cell developmental state line. We combined two axes to one "differentiation index coordinate", and used it to estimate projection positions of different cancers and normal tissues.

**Figure 8:**
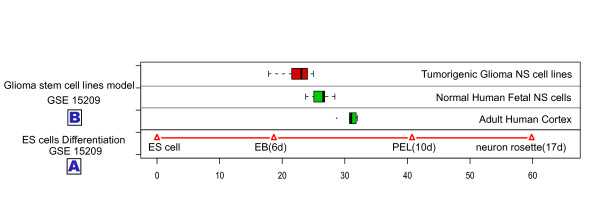
**Human Embryonic Stem cells differentiation state line distinguished distinct temporal temporal distributions of human brain samples and neuron cell lines**. (A) Projection positions of Human Embryonic Stem cells differentiation time-course sample on Cell developmental state line (dataset GSE15209). The ES cells sample was set as origin point; other projection positions were normalized by ES cells sample. (B) Distributions of projection positions of human cortex tissue, normal fetal neuron stem cells and tumorigenic glioma neuron stem cells. Green represents normal tissue and cells. Red represents tumorigenic neuron stem cells.

First we tested the accuracy of the line. The GSE 15209 dataset, which contains expression profiles of normal adult cortex samples, fetal neural stem cells and tumorigenic glioma neural stem cells, was projected onto the "neuronal" cell developmental state line. Compared to adult cells, fetal neural stem cells (f-NS cells), were as expected, more like embryonic stem cells (Figure 8). Surprisingly, tumorigenic glioma neural stem cells (t-g-NS cells) showed the greatest similarity to ES cells at the developmental state level. These results suggest that the tumorigenesis process of glioma tumors may share commons to tumorigenesis in liver and ovary.

Then the "differentiation-index coordinates" was used to detect cell state of four human tumor expression data (GSE7305, GSE4107, GSE5674, and GSE18520) containing normal tissue samples and diseased tissue samples (endometrium, colon, breast, and ovary, respectively) from the GEO (Gene Expression Omnibus). Moreover, we selected expression data from GSE2109 (obtained from the IGC Expression Project for Oncology (expO)), which contains expression data from more than 2000 tumor samples derived from more then one hundred tissues. Tumor samples originating from the breast, colon, endometrium and ovary were individually estimated by differentiation-index coordinates, and the results were shown according to tissue.

**Figure 9:**
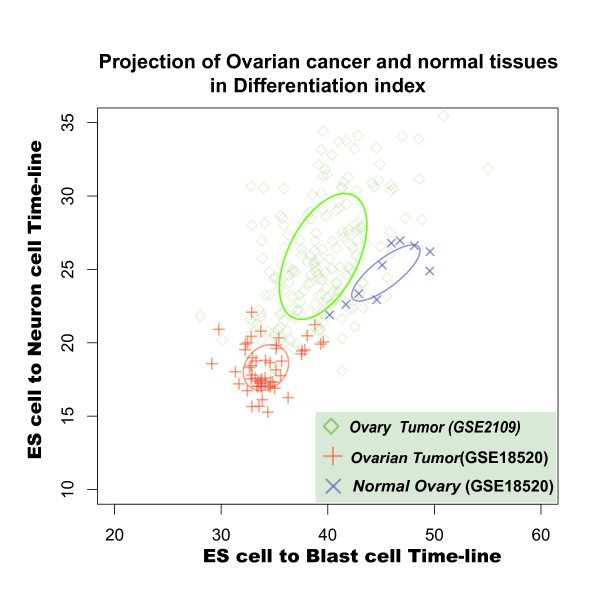
**Distinct cell states of human ovary normal and tumor samples in Differentiation-index Coordinate**. Ovary cell state distribution of normal and tumor samples in Differentiation-index Coordinate based on Human Embryonic Stem cells differentiations state lines. Blue: normal ovary tissues in dataset GSE 18520; Red: ovarian tumor samples in dataset GSE 18520; and Green: ovary cancer samples in dataset GSE 2109.

**Figure 10:**
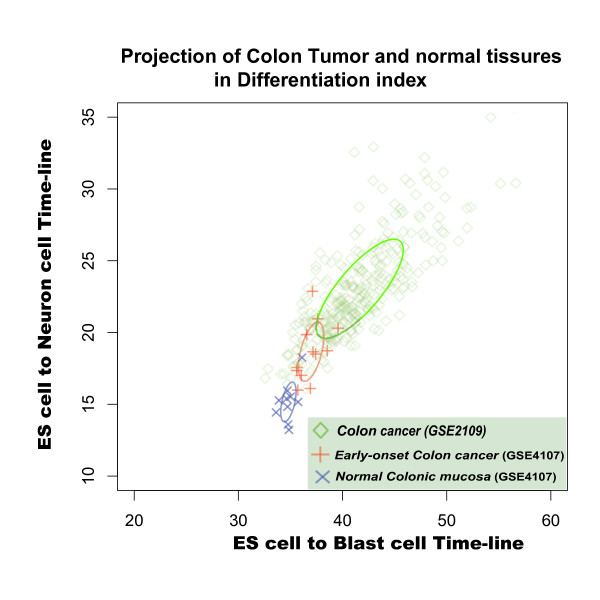
**Distinct cell state of human normal colon and tumor samples in Differentiation-index Coordinate**. Cell state distribution of normal colonic mucosa and early-onset colon cancer samples in Differentiation-index Coordinate based on Human Embryonic Stem cells differentiations estimating distinct. Blue: normal colonic mucosa sample in dataset GSE 4107; Red: early-onset colon cancer samples in dataset GSE 4107; and Green: colon cancer samples in dataset GSE 2109.

In differentiation-index coordinates, ovary tumors, endometriosis, and colorectal tumors were easily distinguished from their corresponding normal tissues. Moreover, ovary tumor samples (dataset GSE18520), tended to resemble ES cells (Figure 9), suggesting that ovary cells become "developmental younger" during tumorigenesis. Colon tumors and endometriosis (datasets GSE7305 and GSE4107) tended to be located at same distance from ES cell state in the differentiation-index coordinates (Figure 10 and Figure S5). Interestingly, the colon cancer seems show a negative correlation between malignance and differentiation: the distribution of projection positions follows the progression from normal tissue to early-onset colon cancer to malignant colon cancer (Figure 10). Such negative correlation in the differentiation-index coordinates suggested that, in a cell state space, the real colon cell developmental state lines may have a opposite direction relative to the direction of the two cell developmental state lines we used here, namely cell-derived neural and blast cell differentiation. (Unfortunately, we have not found any suitable data to calculate the cell developmental state line of the colon.) These results strengthened solid our assumption that the directions of different tissue development indicate the discrete distributions of attractors in cell state space. Like a man walking down from a mountain peak, there are many paths with different directions in cellular development. If we observe development process from an appropriate viewpoint, we may finally understand how many roads must a cell walk down, before we call it a terminal differentiation cell (Additional File 1).

In the case of breast cancer, the tumor and normal breast tissue occupied overlapping positions in the differentiation-index coordinates (Figure S6). The fact that it was not possible to distinguish tumor samples from normal samples by differentiation-index coordinate approach may be due to that tumorigenesis in the breast may not be related to the developmental pathway, or the breast developmental state line may be perpendicular to the "neuronal" and "blast" cell differentiation state lines. Moreover, a recently published comprehensive genome-level catalogue of breast tumors [21] revealed the complexity of breast tumors. Such complexities at genome level may also influence the accuracy of cell developmental state line.

## Discussion

ES cell differentiation is a good model for studying the process of development. However, successful cell differentiation under in vitro conditions is only possible in a limited number of cell types. The two human ES cells developmental state lines developed here, based on in vitro datasets, represent only two of the possible ES cell differentiation directions, and thus cannot fully represent the complete ES cell differentiation process under in vivo conditions. The work presented here provides an approach forwards understanding the relationships among distinct differentiation directions in cell state space, of which microarray space is only one subspace. The increasing availability of high-throughput proteomic and epigenetic data and other measures of cell properties will make it possible to investigate other dimensions.

Theoretically, each type of differentiated cell has its unique differentiation direction. Even a single cellular activity can be considered to have a unique direction. Many different directions exist in the whole process of tissue development and cell differentiation, and these different directions reflect distinct aspects of cell properties. Here, we selected the changing tendency of gene expression pattern over time to represent the differentiation trajectory. In the embryonic development process, the diversity of cells increases continuously with cell differentiation. However, it is difficult to describe the relationships among the growing number of cell types, and how the genome facilitates the generation of stable and distinct cell types in the development process is still not clear.

Assuming that differentiation processed follow a strict order, using a time scale should be useful for estimating changes in cell state throughout the differentiation process. The cell developmental state lines generated by time-ordered linear model were able to accurately order different developmental stages in different tissue types. Interestingly, our results did not only suggest that tumorigenesis can be measured by "developmental state lines", but also suggested the possibility all directions of ES cells differentiation can be described. It has been reported that some genes whose expression is significantly altered during tumorigenesis may also play key roles in developmental process [22-24]. Our approach may help to understand the relationship between tumorigenesis and cell differentiation in greater detail.

Tumorigenesis is a complex process. That tumorigenesis shares many similar characteristics with embryonic cell development implies that they have a close, though poorly understood relationship. The "cancer attractor" model presents a new and integrated perspective for viewing these two processes. Constructing cell developmental state lines is an attempt to observe and describe differentiation trajectories in cell state space from another perspective. Our result indicated that even in large scale discrimination, some kinds of tumor showed the same moving tendency at timescale. Although theoretically there are many "cancer attractors" surrounding normal cell state, in realty the number of these "attractors" may be limited to a very small range.

The time-ordered linear model constructed here is an attempt to use a linear trajectory to describe tissue development and cell differentiation processes. Not only it is a novel method to analyze the time-course expression profiles, but by, primarily defining a biological meaning to the mathematical model, the approach also supplies a different viewpoint to traditional methods which mainly emphasize the mathematical characteristics. The time-ordered linear model is a simplified model for calculating lines to represent development trajectories, and some limitations are still need to be addressed. The calculation of this liner model depends on a given order of sample points, and disordered sample points would generate fake cell development state lines. Moreover, in this work, we used the average of several repeats at each time points to calculate the cell development state line. Such an approach partly limits the robustness and increases the sensitivity to noise. In the future, replacing the averaged points by a may overcome these weaknesses.

However, we cannot expect such a simple model to reflect all the details of development, to fit all expression data, or even to distinguish all types of cancer in cell status space. Rather, its function is simply to transform cell differentiation expression profiles to a line in keeping with the natural temporal properties of gene expression during the development processes. The real development trajectories in cell status space are much too complex to be modeled computationally at present. We have therefore started with a simple "line," which captures the basic progression of development from one perspective. If the approach works, it could serve as a basis for further construction of more realistic, comprehensive, and predictive models of cell state space. To draw developmental trajectories in cell state space accurately requires being able to describe cell state from the perspective of all of cell features, including its transcriptome, epigenetic map, and proteome. Understanding the trajectories in cell status space will help reveal the mysteries of embryonic development.

## Conclusion

By primarily defining a biological meaning to a mathematical model, we designed the time-ordered linear model which can capture temporal properties of development process, and drew the linearly projected development trajectories in a cell state space. Meanwhile, it reflected the change of gene expression from a developmental timescale perspective. By applying this model to measure tumors of different tissues, we found that different developmental states appeared during tumorigenesis.

## Methods

### Construction of our time-ordered linear model

In N dimensional space, T vectors which are unlooped and head-to-tail jointed have many angle-bisectors when **N >> T**. In order to unique our linear model, we selected the angle-bisector which maximized the projection of each vectors (Additional file 2), and we named it "co-bisector". Naturally, we selected co-bisector of a series vectors to indicate their main moving tendency. The co-bisector well suited our requirement of a linear model to represent tissue development and cell differentiation processes.

*X *is a *n *× *t *matrix, which represents expression data containing *n *genes measured at *t *time-points, in which *X_j _*represents the expression profile in time point *j *and the expression score of gene *i *in time points *j *is *x_ij_*.

(1) $$\left(X_{1},X_{2}\cdots X_{t}\right)=\left(\begin{array}{ccc} x_{11} & \ldots & x_{1t}\\ \vdots & \ddots & \vdots \\ x_{n1} & \cdots & x_{nt} \end{array}\right)$$

For the purpose of preserving the strict order of the projected points of *X_i_*, *i*∈[1,*t*] on the projected line, we firstly generated *(t-1) *vectors $\vec{X_{t}X_{t+1}},i\in [1,t-1]$. The vectors are given by:

(2) $$\begin{array}{l} \vec{X_{i}X_{\left(i+1\right)}}=X_{\left(i+1\right)}-X_{i}\\ \;\;=\left(\begin{array}{c} x_{1(i+1)}\\ \vdots \\ x_{n(i+1)} \end{array}\right)-\left(\begin{array}{c} x_{1i}\\ \vdots \\ x_{ni} \end{array}\right)\\ \;\;\;i\in \left[1,t-1\right] \end{array}$$

Then, we defined the co-bisector as $\vec{e_{all}}$. $\vec{X_{i}X_{\left(i+1\right)}}$ and the co-bisector $\vec{e_{all}}$ should satisfy the equation below:

(3) $$\begin{array}{c} \langle \vec{e_{all}},\vec{X_{i}X_{\left(i+1\right)}}\rangle =\left\|\vec{e_{all}}\right\|•\left\|\vec{X_{i}X_{\left(i+1\right)}}\right\|•\cos\theta \\ i\in [1,t-1] \end{array}$$

Naturally, after points of *X_i_*, *i*∈[1,*t*] were projected onto the bisector of $\vec{X_{t}X_{t+1}},i\in [1,t-1]$, the projection of points still retain their orders. Among all bisectors which could preserve the distance ratio of these sample points by projection, the bisector $\vec{e_{all}{}^{*}}$ locates in the N-dimensional subspace which determined by $\vec{X_{t}X_{t+1}},i\in [1,t-1]$, and it has the longest length. Thus, the optimized $\vec{e_{all}{}^{*}}$ could be represented as the linear combination of $\vec{X_{t}X_{t+1}},i\in [1,t-1]$:

(4) $$\vec{e_{all}}=\sum_{i=1}^{t-1}(a_{i}•\vec{X_{i}X_{\left(i+1\right)}})\;a_{i}\in R$$

To simplify our calculations, we set $\left\|\vec{e_{all}}\right\|$ as 1:

(5) $$\left\|\vec{e_{all}}\right\|=1$$

With equation (2)(3)(4)(5), the parameters *a_i _*and *θ*, and vector $\vec{e_{all}{}^{*}}$ were determined.

### Projecting expression data on the cell developmental state line

The co-bisector $\vec{e_{all}{}^{*}}$ is the line which represents the temporal properties of the differentiation curve, so we call this co-bisector the "cell developmental state line". This cell developmental state line reflects the degree of cell state change during the cell differentiation process, from pluripotency to the fully differentiated cell state, and can be used to assess the differentiation stage of experimental samples. When the expression profiles of the samples are projected, the projection position Pi of each sample is calculated by:

(6) $$\begin{array}{c} P_{i}={X^{\prime }}_{i}\cdot \vec{e_{all}{}^{*}}=\left(x_{1i},\cdots ,x_{ni}\right)•\left(\begin{array}{c} e_{1}\\ \vdots \\ e_{n} \end{array}\right)\\ i\in \left[1,t-1\right] \end{array}$$

### Datasets

We applied our time-ordered linear model to analyze time-course experimental expression profiles obtained from the Gene Expression Omnibus http://www.ncbi.nlm.nih.gov/geo/ for mouse liver, ovary and lung tissue development, and for human ES cell differentiation. We constructed tissue-specific "cell developmental state lines" to represent developmental trajectories and used them to estimate the relationship between tumor tissues and normal tissue development over time. To increase the resolution, we also constructed "differentiation-index coordinates", which consisted of two different "cell developmental state lines" based on the human ES cell differentiation datasets. We used the datasets listed in Table S3 to generate mouse tissue-specific developmental cell developmental state lines. We then used the model to estimate the relationship between the tumor and normal tissue expression profiles listed in Table S4. Human ES cell differentiation cell developmental state lines were generated using the datasets listed in Table S5, and then human cell developmental state line were used to assess the expression profiles of human tumors listed in Table S6.

## Competing interests

The authors declare that they have no competing interests.

## Authors' contributions

BZ collected data, designed and constructed the model, analyzed data, and drafted the manuscript. BBC participated in data collection and analysis. XPZ conceived the study, constructed the model, supervised the study and manuscript. TW participated in designing the model. ZYX helped to draft the manuscript. RSC designed and supervised all study. All authors read and approved the final manuscript

## Supplementary Material

Additional file 1 **Changing our viewpoints**. Once changing viewpoints of observation development, the structure of cell differentiation forked tree will be different. Click here for file

Additional file 2 **Maximization of the projection of each vectors on co-angle-bisector**. Maximization of the projection of each vectors on co-angle-bisector. Click here for file
