# Supplementary material for: Estimating developmental states of tumors and normal tissues using a linear time-ordered model
Source: BMC Bioinformatics. 2011 Feb 11;12:53. doi: 10.1186/1471-2105-12-53 (PMC3223864; doi:10.1186/1471-2105-12-53)
Supplement: Additional file 1 — Changing our viewpoints. Once changing viewpoints of observation development, the structure of cell differentiation forked tree will be different. [file 1471-2105-12-53-S1.PDF]

Changing our viewpoints

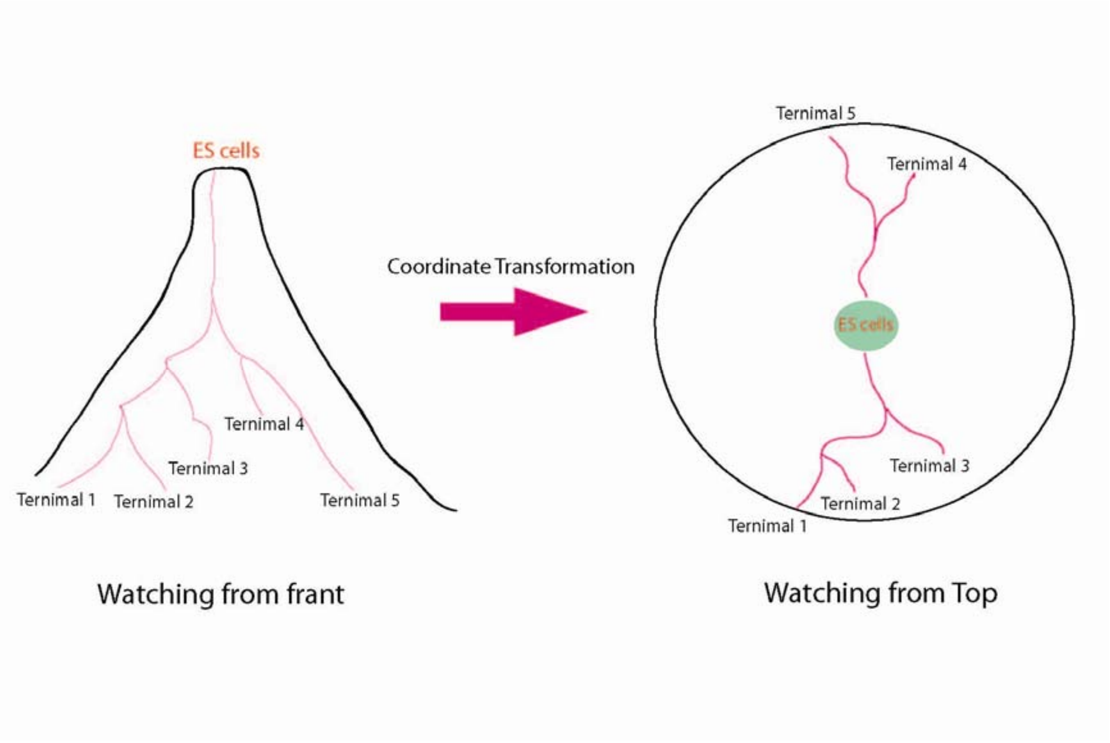

The details of ES cells differentiation to all kinds of terminal differentiated cell are still not clear.

(In Waddington's epigenetic landscape. A marble (the cell) rolls down to the valleys, hence, is "attracted" by them and stays in them. Thus, Waddington's valleys are formally equivalent to Stuart Kauffman's attractors that can exist in a distinct subclass of complex, discrete-valued model gene networks. Kauffman proposed in 1969 that attractors represent cell types. The basins of attraction 'drain' the valleys, that is, an attractor "attracts" less stable states in its neighborhood, hence forcing them to spontaneously implement the gene expression profile S(t) represented by the attractor's position the state space.)

Cited form webpage of Prif. Sui Huang (http://www.childrenshospital.org/research/huanglab/)

The structure of cell differentiation forked tree (left) can explain the affiliation between each kinds cell types (Left). However, when we try to use a direction of A tissue development to measure cell state of B tissue, the direction of B tissue development must be considered. For meeting this requirement, we need to change our viewpoint to observe the process of cell differentiation.
