# Supplementary material for: Estimating developmental states of tumors and normal tissues using a linear time-ordered model
Source: BMC Bioinformatics. 2011 Feb 11;12:53. doi: 10.1186/1471-2105-12-53 (PMC3223864; doi:10.1186/1471-2105-12-53)
Supplement: Additional file 2 — Maximization of the projection of each vectors on co-angle-bisector. Maximization of the projection of each vectors on co-angle-bisector. [file 1471-2105-12-53-S2.PDF]

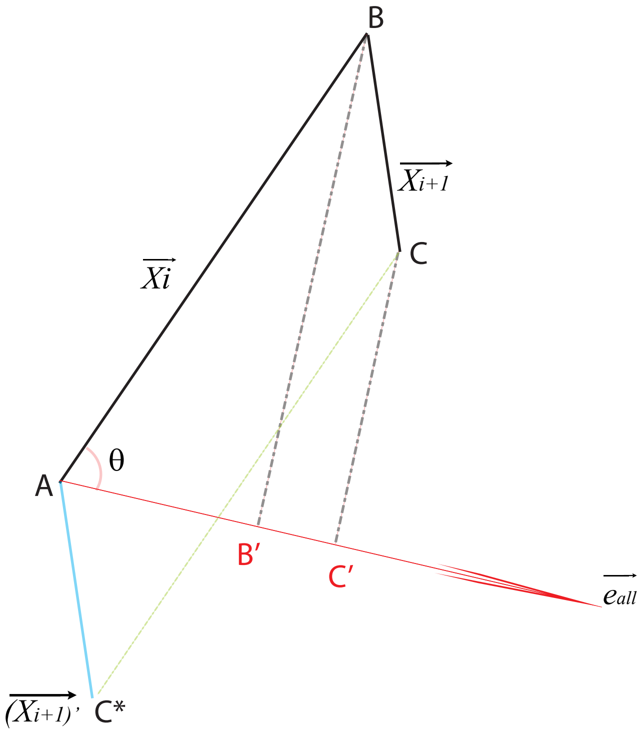

B
$\overrightarrow{X_{i+1}}$
C
$\overrightarrow{Xi}$
θ
A
B′
C′
$\overrightarrow{e_{all}}$
$\overrightarrow{(X_{i+1})'}$
C*

Vector $\overrightarrow{X_i}$ and $\overrightarrow{X_{i+1}}$ existing in a 3-D space, represent a cell departed form state A , bypassing state B, finally reached to state C. First we transform location of $\overrightarrow{X_{i+1}}$ to $\overrightarrow{(X_{i+1})'}$, then we get the angle $\angle\theta$, then generate one angle-bisector $\overrightarrow{e_{all}}$, and angle $\angle\theta = \frac{1}{2}\angle BAC^*$. On this angle-bisector, $\overrightarrow{AB'}$ and $\overrightarrow{B'C'}$ are projections of Vector $\overrightarrow{AB}$ and $\overrightarrow{BC}$.

$$\left\|\overrightarrow{AB'}\right\| = \left\|\overrightarrow{AB}\right\| \times \cos(\theta)$$

$$\left\|\overrightarrow{B'C'}\right\| = \left\|\overrightarrow{BC}\right\| \times \cos(\theta)$$

However, in this 3-D space, there exist a plane $\overrightarrow{e_{all}} \wedge \overrightarrow{e_{all^*}}$ which is perpendicular to the plane ABC; each line passing point A are a angle-bisector of $\angle BAC^*$, all of them meeting our requirement. Obviously, when the included angle θ^* is minimized, the projection $\overrightarrow{AB'^*}$ and $\overrightarrow{B'C'^*}$ are maximized, at this time, the maximized angel-bisector is uniquely determined by intersection of plane $\overrightarrow{e_{all}} \wedge \overrightarrow{e_{all^*}}$ and plane ABC. When the dimensions of this space is over 3, the maximized angel-bisector is uniquely determined by intersection of all angle-bisector plane $[\overrightarrow{e_{all(i)}} \wedge \overrightarrow{e_{all(i)^*}}], i \in [1, t-1]$.

So, when the angle-bisector exist in the subspace which is determined by the parent vector, the projection length of each vector is maximized.

Proof: As we know, there exists a e_{all}^*

$$e_{all}^* = \sum a_i \frac{X_i}{\|X_i\|} \qquad a_i \in R \quad i \in (1, n)$$

$$\|e_{all}^*\| = 1$$

$$e_{all}^* \cdot \frac{X_i}{\|X_i\|} = \cos\theta^* \qquad i \in (1, n)$$

Let assume there is an e_{all} satisfies that

$$\|e_{all}\| = 1$$

$$e_{all} \cdot \frac{X_i}{\|X_i\|} = \cos\theta \qquad i \in (1, n)$$

$$\cos\theta > \cos\theta^*$$

Then

$$e_{all}^* \cdot e_{all} = \sum a_i \frac{X_i}{\|X_i\|} \cdot e_{all}$$

$$= \sum a_i \cos\theta$$

$$> \sum a_i \cos\theta^*$$

$$= \sum a_i \frac{X_i}{\|X_i\|} \cdot e_{all}^* = e_{all}^* \cdot e_{all}^* = 1$$

This is impossible. So there esists no e_{all} that can satisfied $\cos\theta > \cos\theta^*$. Thus, e_{all}^* is the longest bisector.
